# Di-μ-aqua-bis­(μ-pyridazine-4-carboxyl­ato-κ^2^ *N*:*N*′)bis­[triaqua­(pyridazine-4-carboxyl­ato-κ^2^ *O*,*O*′)lead(II)] dihydrate

**DOI:** 10.1107/S1600536809039658

**Published:** 2009-10-03

**Authors:** Wojciech Starosta, Janusz Leciejewicz

**Affiliations:** a Institute of Nuclear Chemistry and Technology, ul.Dorodna 16, 03-195 Warszawa, Poland

## Abstract

The structure of the title compound, [Pb_2_(C_5_H_3_N_2_O_2_)_4_(H_2_O)_6_]·2H_2_O, is composed of dimeric mol­ecules in which two symmetry-related Pb^2+^ ions are bridged by a pair of two pyridazine-4-carboxyl­ate ligand mol­ecules *via* both heterocyclic N atoms and two water O atoms. Each Pb^2+^ ion is also coordinated by two carboxyl­ate O atoms and three water O atoms, leading to a highly irregular coordination polyhedron around Pb^2+^. The dimers are inter­connected by hydrogen bonds between coordinated and uncoordinated water mol­ecules and the carboxyl­ate O atoms. O—H⋯N inter­actions are also present.

## Related literature

For the crystal structure of pyridazine-4-carboxylic acid hydro­chloride, see: Starosta & Leciejewicz (2008 ▶). Centrosymmetric dimeric mol­ecules were reported in the structure of a calcium(II) complex with pyridazine-3-dicarboxyl­ate and water ligands (Starosta & Leciejewicz, 2007 ▶) and an uranyl complex with the same ligands (Leciejewicz & Starosta, (2009 ▶). Each dimer shows a different bridging mode.
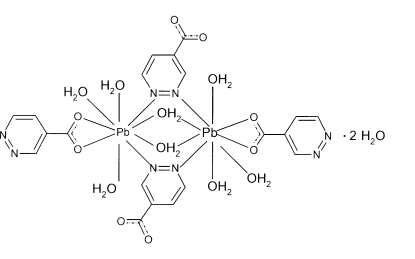


## Experimental

### 

#### Crystal data

[Pb_2_(C_5_H_3_N_2_O_2_)_4_(H_2_O)_6_]·2H_2_O
*M* *_r_* = 1086.92
Triclinic, 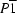

*a* = 7.0762 (14) Å
*b* = 9.2967 (19) Å
*c* = 12.830 (3) Å
α = 92.05 (3)°
β = 105.13 (3)°
γ = 102.85 (3)°
*V* = 790.4 (3) Å^3^
*Z* = 1
Mo *K*α radiation
μ = 10.73 mm^−1^
*T* = 293 K
0.35 × 0.18 × 0.03 mm

#### Data collection

Kuma KM-4 four-circle diffractometer
Absorption correction: analytical (*CrysAlis RED*; Oxford Diffraction, 2008 ▶) *T*_min_ = 0.254, *T*_max_ = 0.762
4862 measured reflections
4512 independent reflections
3958 reflections with *I* > 2σ(*I*)
*R*_int_ = 0.016
3 standard reflections every 200 reflections intensity decay: 1.2%

#### Refinement

*R*[*F* ^2^ > 2σ(*F* ^2^)] = 0.045
*wR*(*F* ^2^) = 0.122
*S* = 1.06
4512 reflections
253 parameters
15 restraints
H atoms treated by a mixture of independent and constrained refinement
Δρ_max_ = 5.63 e Å^−3^
Δρ_min_ = −3.77 e Å^−3^

### 

Data collection: *KM-4 Software* (Kuma, 1996 ▶); cell refinement: *KM-4 Software*; data reduction: *DATAPROC* (Kuma, 2001 ▶); program(s) used to solve structure: *SHELXS97* (Sheldrick, 2008 ▶); program(s) used to refine structure: *SHELXL97* (Sheldrick, 2008 ▶); molecular graphics: *SHELXTL* (Sheldrick, 2008 ▶); software used to prepare material for publication: *SHELXL97*.

## Supplementary Material

Crystal structure: contains datablocks I, global. DOI: 10.1107/S1600536809039658/bv2128sup1.cif

Structure factors: contains datablocks I. DOI: 10.1107/S1600536809039658/bv2128Isup2.hkl

Additional supplementary materials:  crystallographic information; 3D view; checkCIF report

## Figures and Tables

**Table 1:** Hydrogen-bond geometry (Å, °)

*D*—H⋯*A*	*D*—H	H⋯*A*	*D*⋯*A*	*D*—H⋯*A*
O4—H41⋯O12^i^	0.86 (2)	1.93 (2)	2.736 (6)	154 (4)
O4—H42⋯O12^ii^	0.87 (2)	1.92 (4)	2.754 (6)	161 (11)
O1—H11⋯O5	0.86 (2)	2.08 (3)	2.943 (9)	174 (10)
O1—H12⋯O11^iii^	0.86 (2)	2.09 (6)	2.849 (7)	146 (9)
O5—H51⋯N22^iv^	0.86 (2)	2.23 (5)	3.013 (8)	151 (9)
O5—H52⋯O21^v^	0.87 (2)	2.12 (6)	2.897 (8)	149 (10)
O3—H31⋯O12^vi^	0.86 (2)	2.09 (7)	2.819 (7)	142 (10)
O3—H32⋯N21^iv^	0.86 (2)	2.01 (6)	2.794 (7)	151 (10)
O2—H21⋯O5^vii^	0.86 (2)	2.13 (5)	2.906 (8)	150 (9)
O2—H22⋯O11^ii^	0.86 (2)	2.07 (4)	2.891 (7)	159 (9)

Symmetry codes: (i) 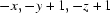; (ii) 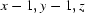; (iii) 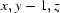; (iv) 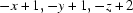; (v) 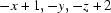; (vi) 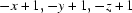; (vii) 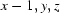.

**Fig. 1.**
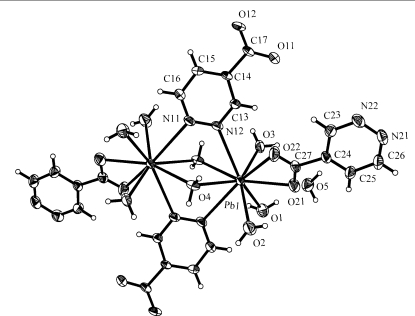
A dimer of (1) with atom labelling scheme and 50% probability displacement ellipsoids.

**Fig. 2.**
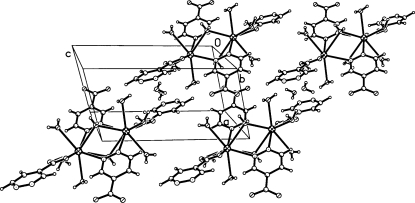
Packing diagram of the structure.

## supplementary crystallographic information

### Comment

The structure of the title compound (I) is built of dimeric molecules (Fig.1) in which two symmetry related, nine-coordinate Pb^2+^ ions are chelated by two pairs of pyridazine-4-carboxylate anions *via* their both hetero-ring atoms, each pair showing different chelating mode: one uses both its hetero-ring N atoms to bridge the metal ions, its deprotonated carboxylate O atoms are left inactive in coordination, the other coordinates the Pb^2+^ions only *via* carboxylate groups which act as bidentate. In addition, the Pb^2+^ ions are bridged by a pair of water molecules. Three coordinated water O atoms complete the coordination enviroment of Pb^2+^ ions. The coordination geometry around a metal ion is highly irregular. Pyridazine rings are planar with r.m.s. 0.0081 (2)Å (ligand 1) and 0.0030 (2)Å (ligand 2). The coordinated (C27/O21/O22) and non-coordinated (C17/O11/O12) carboxylic groups make dihedral angles of 5.6 (1)° and 11.9 (1)° with their respective pyridazine rings. A packing diagram of (I) displayed in Fig.2 shows how the dimers are linked by a network of hydrogen bonds. Their relevant geometrical parameters are listed in Table 1.

### Experimental

2 Mmol of pyridazine-4-carboxylic acid were dissolved in 100 ml of hot water and boiled for two hours with small excess of Pb(OH)_2_. After cooling to room temperature the mixture was filtered and left to crystallize. Few days later colorless single crystals were found in the mother liquid. They were separated, washed with cold ethanol and dried in air.

### Refinement

H atoms attached to pyridazine-ring C atoms were positioned geometrically and refined with a riding model using AFIX43 instruction. The positions of water H atoms were initially located from Fourier maps and refined isotropically with restraints on O—H distance (0.86 Å) and H—O—H angle.

### Crystal data

**Table:**

[Pb_2_(C_5_H_3_N_2_O_2_)_4_(H_2_O)_6_]·2H_2_O	*Z* = 1
*M**_r_* = 1086.92	*F*(000) = 516
Triclinic, *P*1	*D*_x_ = 2.283 Mg m^−^^3^
*a* = 7.0762 (14) Å	Mo *K*α radiation, λ = 0.71073 Å
*b* = 9.2967 (19) Å	Cell parameters from 25 reflections
*c* = 12.830 (3) Å	θ = 6–15°
α = 92.05 (3)°	µ = 10.73 mm^−^^1^
β = 105.13 (3)°	*T* = 293 K
γ = 102.85 (3)°	Plate, colourless
*V* = 790.4 (3) Å^3^	0.35 × 0.18 × 0.03 mm

### Data collection

**Table:**

Kuma KM-4 four-circle diffractometer	3958 reflections with *I* > 2σ(*I*)
Radiation source: fine-focus sealed tube	*R*_int_ = 0.016
graphite	θ_max_ = 30.2°, θ_min_ = 1.7°
Profile data from ω/2θ scans	*h* = 0→9
Absorption correction: analytical (*CrysAlis RED*; Oxford Diffraction, 2008)	*k* = −13→12
*T*_min_ = 0.254, *T*_max_ = 0.762	*l* = −18→16
4862 measured reflections	3 standard reflections every 200 reflections
4512 independent reflections	intensity decay: 1.2%

### Refinement

**Table:**

Refinement on *F*^2^	Primary atom site location: structure-invariant direct methods
Least-squares matrix: full	Secondary atom site location: difference Fourier map
*R*[*F*^2^ > 2σ(*F*^2^)] = 0.045	Hydrogen site location: inferred from neighbouring sites
*wR*(*F*^2^) = 0.122	H atoms treated by a mixture of independent and constrained refinement
*S* = 1.06	*w* = 1/[σ^2^(*F*_o_^2^) + (0.1029*P*)^2^] where *P* = (*F*_o_^2^ + 2*F*_c_^2^)/3
4512 reflections	(Δ/σ)_max_ = 0.002
253 parameters	Δρ_max_ = 5.63 e Å^−^^3^
15 restraints	Δρ_min_ = −3.77 e Å^−^^3^

### Special details

**Table:**

Geometry. All e.s.d.'s (except the e.s.d. in the dihedral angle between two l.s. planes) are estimated using the full covariance matrix. The cell e.s.d.'s are taken into account individually in the estimation of e.s.d.'s in distances, angles and torsion angles; correlations between e.s.d.'s in cell parameters are only used when they are defined by crystal symmetry. An approximate (isotropic) treatment of cell e.s.d.'s is used for estimating e.s.d.'s involving l.s. planes.
Refinement. Refinement of *F*^2^ against ALL reflections. The weighted *R*-factor *wR* and goodness of fit *S* are based on *F*^2^, conventional *R*-factors *R* are based on *F*, with *F* set to zero for negative *F*^2^. The threshold expression of *F*^2^ > σ(*F*^2^) is used only for calculating *R*-factors(gt) *etc*. and is not relevant to the choice of reflections for refinement. *R*-factors based on *F*^2^ are statistically about twice as large as those based on *F*, and *R*- factors based on ALL data will be even larger.

### Fractional atomic coordinates and isotropic or equivalent isotropic displacement parameters (Å^2^)

**Table:**

	*x*	*y*	*z*	*U*_iso_*/*U*_eq_	
Pb1	0.11555 (3)	0.048890 (18)	0.675973 (14)	0.02300 (9)	
O11	0.4666 (8)	0.6974 (5)	0.6607 (4)	0.0360 (10)	
O4	−0.2317 (7)	0.0086 (5)	0.5150 (4)	0.0309 (9)	
O21	0.2153 (8)	0.1496 (5)	0.8788 (4)	0.0387 (11)	
O1	0.3206 (8)	−0.1283 (6)	0.7946 (4)	0.0395 (11)	
H11	0.430 (8)	−0.075 (11)	0.838 (5)	0.047*	
H12	0.347 (11)	−0.155 (10)	0.736 (4)	0.047*	
N12	0.1246 (8)	0.2794 (5)	0.5304 (4)	0.0265 (9)	
N11	0.0425 (8)	0.2525 (5)	0.4237 (4)	0.0280 (10)	
O22	0.0922 (10)	0.3005 (6)	0.7692 (4)	0.0452 (13)	
O12	0.4408 (7)	0.7751 (4)	0.4972 (4)	0.0331 (9)	
C17	0.4044 (8)	0.6811 (6)	0.5601 (5)	0.0247 (10)	
C14	0.2716 (7)	0.5309 (5)	0.5091 (4)	0.0208 (9)	
C13	0.2351 (9)	0.4143 (6)	0.5710 (5)	0.0249 (10)	
H13	0.2916	0.4317	0.6458	0.030*	
C15	0.1807 (9)	0.5029 (6)	0.3995 (5)	0.0273 (11)	
H15	0.1940	0.5769	0.3532	0.033*	
C16	0.0682 (10)	0.3600 (7)	0.3606 (5)	0.0309 (12)	
H16	0.0079	0.3389	0.2863	0.037*	
C24	0.2220 (9)	0.3860 (6)	0.9548 (5)	0.0273 (11)	
C27	0.1684 (10)	0.2701 (6)	0.8588 (5)	0.0297 (11)	
O5	0.7080 (10)	0.0331 (7)	0.9439 (4)	0.0471 (13)	
H51	0.753 (16)	0.123 (4)	0.975 (6)	0.057*	
H52	0.682 (16)	−0.024 (7)	0.993 (5)	0.057*	
N21	0.3150 (10)	0.6062 (7)	1.1197 (5)	0.0385 (13)	
N22	0.2595 (11)	0.6394 (6)	1.0184 (6)	0.0391 (13)	
C25	0.2804 (11)	0.3556 (7)	1.0582 (5)	0.0342 (13)	
H23	0.2907	0.2606	1.0748	0.041*	
C23	0.2131 (11)	0.5332 (7)	0.9376 (5)	0.0325 (12)	
H25	0.1731	0.5571	0.8667	0.039*	
C26	0.3247 (13)	0.4707 (9)	1.1396 (6)	0.0396 (15)	
H26	0.3631	0.4503	1.2115	0.048*	
O3	0.4919 (9)	0.1749 (7)	0.7070 (4)	0.0449 (12)	
O2	−0.1694 (9)	−0.0786 (7)	0.7638 (5)	0.0490 (14)	
H41	−0.277 (8)	0.082 (6)	0.533 (5)	0.07 (3)*	
H42	−0.316 (12)	−0.072 (6)	0.521 (10)	0.07 (3)*	
H31	0.529 (18)	0.231 (10)	0.661 (6)	0.07 (4)*	
H32	0.557 (16)	0.215 (11)	0.771 (3)	0.08 (4)*	
H21	−0.172 (17)	−0.018 (9)	0.815 (6)	0.05 (3)*	
H22	−0.259 (10)	−0.160 (6)	0.740 (7)	0.04 (2)*	

### Atomic displacement parameters (Å^2^)

**Table:**

	*U*^11^	*U*^22^	*U*^33^	*U*^12^	*U*^13^	*U*^23^
Pb1	0.02442 (13)	0.01967 (12)	0.02294 (13)	0.00112 (8)	0.00730 (9)	−0.00286 (7)
O11	0.040 (2)	0.026 (2)	0.032 (2)	−0.0024 (18)	0.0027 (19)	−0.0031 (17)
O4	0.029 (2)	0.0177 (18)	0.044 (3)	0.0019 (15)	0.0105 (19)	0.0037 (16)
O21	0.049 (3)	0.029 (2)	0.035 (2)	0.010 (2)	0.008 (2)	−0.0061 (18)
O1	0.041 (3)	0.039 (3)	0.039 (3)	0.012 (2)	0.010 (2)	−0.003 (2)
N12	0.031 (2)	0.0161 (19)	0.032 (2)	0.0016 (17)	0.012 (2)	0.0006 (17)
N11	0.032 (2)	0.0169 (19)	0.032 (2)	0.0005 (17)	0.009 (2)	−0.0032 (17)
O22	0.069 (4)	0.039 (3)	0.025 (2)	0.016 (3)	0.007 (2)	−0.0038 (19)
O12	0.036 (2)	0.0166 (17)	0.046 (3)	−0.0025 (16)	0.016 (2)	0.0023 (16)
C17	0.022 (2)	0.015 (2)	0.036 (3)	0.0028 (17)	0.009 (2)	−0.0044 (18)
C14	0.019 (2)	0.0133 (19)	0.031 (3)	0.0033 (16)	0.0100 (19)	−0.0011 (17)
C13	0.025 (2)	0.020 (2)	0.028 (3)	0.0035 (19)	0.006 (2)	0.0020 (19)
C15	0.030 (3)	0.019 (2)	0.033 (3)	0.003 (2)	0.010 (2)	0.006 (2)
C16	0.032 (3)	0.024 (3)	0.030 (3)	0.000 (2)	0.003 (2)	−0.002 (2)
C24	0.032 (3)	0.023 (2)	0.026 (3)	0.005 (2)	0.007 (2)	−0.0055 (19)
C27	0.038 (3)	0.024 (2)	0.027 (3)	0.004 (2)	0.011 (2)	−0.006 (2)
O5	0.064 (4)	0.042 (3)	0.039 (3)	0.016 (3)	0.017 (3)	0.001 (2)
N21	0.040 (3)	0.037 (3)	0.034 (3)	0.004 (2)	0.009 (2)	−0.013 (2)
N22	0.048 (3)	0.025 (2)	0.042 (3)	0.009 (2)	0.010 (3)	−0.005 (2)
C25	0.048 (4)	0.031 (3)	0.025 (3)	0.013 (3)	0.012 (3)	0.001 (2)
C23	0.044 (3)	0.028 (3)	0.027 (3)	0.013 (2)	0.009 (2)	0.003 (2)
C26	0.052 (4)	0.042 (4)	0.024 (3)	0.010 (3)	0.009 (3)	−0.006 (2)
O3	0.043 (3)	0.051 (3)	0.025 (2)	−0.016 (2)	0.008 (2)	−0.007 (2)
O2	0.045 (3)	0.053 (3)	0.042 (3)	−0.012 (2)	0.023 (2)	−0.011 (2)

### Geometric parameters (Å, °)

**Table:**

Pb1—O3	2.578 (6)	C13—H13	0.9300
Pb1—O21	2.593 (5)	C15—C16	1.386 (8)
Pb1—O2	2.638 (6)	C15—H15	0.9300
Pb1—O22	2.647 (5)	C16—H16	0.9300
Pb1—O1	2.688 (6)	C24—C25	1.343 (9)
Pb1—O4	2.707 (5)	C24—C23	1.406 (8)
O11—C17	1.242 (8)	C24—C27	1.517 (8)
O4—H41	0.86 (2)	O5—H51	0.86 (2)
O4—H42	0.87 (2)	O5—H52	0.87 (2)
O21—C27	1.254 (8)	N21—C26	1.308 (10)
O1—H11	0.86 (2)	N21—N22	1.324 (10)
O1—H12	0.86 (2)	N22—C23	1.328 (8)
N12—C13	1.327 (7)	C25—C26	1.390 (9)
N12—N11	1.331 (7)	C25—H23	0.9300
N11—C16	1.320 (8)	C23—H25	0.9300
O22—C27	1.208 (8)	C26—H26	0.9300
O12—C17	1.241 (7)	O3—H31	0.86 (2)
C17—C14	1.514 (7)	O3—H32	0.86 (2)
C14—C15	1.373 (8)	O2—H21	0.86 (2)
C14—C13	1.385 (7)	O2—H22	0.86 (2)
			
O3—Pb1—O21	79.05 (17)	N12—C13—H13	118.0
O3—Pb1—O2	146.72 (18)	C14—C13—H13	118.0
O21—Pb1—O2	70.88 (18)	C14—C15—C16	117.4 (5)
O3—Pb1—O22	85.5 (2)	C14—C15—H15	121.3
O21—Pb1—O22	49.16 (16)	C16—C15—H15	121.3
O2—Pb1—O22	85.5 (2)	N11—C16—C15	123.2 (6)
O3—Pb1—O1	74.2 (2)	N11—C16—H16	118.4
O21—Pb1—O1	71.28 (16)	C15—C16—H16	118.4
O2—Pb1—O1	82.8 (2)	C25—C24—C23	117.0 (5)
O22—Pb1—O1	119.77 (16)	C25—C24—C27	123.0 (6)
O3—Pb1—O4	137.65 (16)	C23—C24—C27	120.1 (6)
O21—Pb1—O4	132.46 (16)	O22—C27—O21	124.6 (6)
O2—Pb1—O4	75.25 (18)	O22—C27—C24	119.1 (6)
O22—Pb1—O4	96.49 (16)	O21—C27—C24	116.3 (6)
O1—Pb1—O4	135.76 (15)	H51—O5—H52	107 (3)
Pb1—O4—H41	100.9 (17)	C26—N21—N22	120.3 (6)
Pb1—O4—H42	110 (8)	N21—N22—C23	119.1 (6)
H41—O4—H42	107 (3)	C24—C25—C26	117.9 (6)
C27—O21—Pb1	93.8 (4)	C24—C25—H23	121.1
Pb1—O1—H11	110 (8)	C26—C25—H23	121.1
Pb1—O1—H12	87 (7)	N22—C23—C24	122.7 (6)
H11—O1—H12	109 (3)	N22—C23—H25	118.7
C13—N12—N11	118.9 (5)	C24—C23—H25	118.7
C16—N11—N12	119.9 (5)	N21—C26—C25	123.0 (7)
C27—O22—Pb1	92.3 (4)	N21—C26—H26	118.5
O12—C17—O11	126.7 (5)	C25—C26—H26	118.5
O12—C17—C14	116.7 (5)	Pb1—O3—H31	120 (8)
O11—C17—C14	116.6 (5)	Pb1—O3—H32	117 (8)
C15—C14—C13	116.5 (5)	H31—O3—H32	109 (4)
C15—C14—C17	122.0 (5)	Pb1—O2—H21	108 (7)
C13—C14—C17	121.5 (5)	Pb1—O2—H22	127 (6)
N12—C13—C14	124.0 (5)	H21—O2—H22	124 (10)

### Hydrogen-bond geometry (Å, °)

**Table:**

*D*—H···*A*	*D*—H	H···*A*	*D*···*A*	*D*—H···*A*
O4—H41···O12^i^	0.86 (2)	1.93 (2)	2.736 (6)	154 (4)
O4—H42···O12^ii^	0.87 (2)	1.92 (4)	2.754 (6)	161 (11)
O1—H11···O5	0.86 (2)	2.08 (3)	2.943 (9)	174 (10)
O1—H12···O11^iii^	0.86 (2)	2.09 (6)	2.849 (7)	146 (9)
O5—H51···N22^iv^	0.86 (2)	2.23 (5)	3.013 (8)	151 (9)
O5—H52···O21^v^	0.87 (2)	2.12 (6)	2.897 (8)	149 (10)
O3—H31···O12^vi^	0.86 (2)	2.09 (7)	2.819 (7)	142 (10)
O3—H32···N21^iv^	0.86 (2)	2.01 (6)	2.794 (7)	151 (10)
O2—H21···O5^vii^	0.86 (2)	2.13 (5)	2.906 (8)	150 (9)
O2—H22···O11^ii^	0.86 (2)	2.07 (4)	2.891 (7)	159 (9)

Symmetry codes: (i) −*x*, −*y*+1, −*z*+1; (ii) *x*−1, *y*−1, *z*; (iii) *x*, *y*−1, *z*; (iv) −*x*+1, −*y*+1, −*z*+2; (v) −*x*+1, −*y*, −*z*+2; (vi) −*x*+1, −*y*+1, −*z*+1; (vii) *x*−1, *y*, *z*.
